# Bedaquiline, pretomanid, linezolid, and moxifloxacin (BPaLM) for multidrug- or rifampin-resistant tuberculosis: a systematic review

**DOI:** 10.36416/1806-3756/e20240295

**Published:** 2024-12-17

**Authors:** Denise Rossato Silva, Flávia Fonseca Fernandes, Juliana Carvalho Ferreira, Wanderley Bernando, Margareth Maria Pretti Dalcolmo, Fernanda Dockhorn Costa Johansen, Fernanda Carvalho de Queiroz Mello

**Affiliations:** 1. Faculdade de Medicina, Universidade Federal do Rio Grande do Sul -UFRGS - Porto Alegre (RS) Brasil.; 2. Departamento de Medicina, Universidade Federal de Catalão, Catalão (GO) Brasil.; 3. Instituto do Coração - InCor - Hospital das Clínicas, Faculdade de Medicina, Universidade de São Paulo, São Paulo (SP) Brasil.; 4. Faculdade de Medicina, Universidade de São Paulo, São Paulo (SP) Brasil.; 5. Centro de Referência Hélio Fraga, Fiocruz, Rio de Janeiro (RJ) Brasil.; 6. Ministério da Saúde, Secretaria em Vigilância em Saúde e Ambiente, Coordenação-Geral de Vigilância da Tuberculose, Micoses Endêmicas e Micobactérias não Tuberculosas, Brasília (DF) Brasil.; 7. Instituto de Doenças do Tórax - IDT - Faculdade de Medicina, Universidade Federal do Rio de Janeiro - UFRJ - Rio de Janeiro (RJ) Brasil.

**Keywords:** Tuberculosis, multidrug-resistant, Antitubercular agents, Diarylquinolines, Linezolid, Moxifloxacin, Nitroimidazoles

## Abstract

**Objective:**

To evaluate the available evidence comparing the use of the bedaquiline, pretomanid, linezolid, and moxifloxacin (BPaLM) regimen for 6 months with that of standard-of-care regimens for patients with multidrug-resistant or rifampin-resistant tuberculosis (MDR/RR-TB).

**Methods:**

This was a systematic review of clinical trials comparing the use of the BPaLM regimen with the standard of care in patients with MDR/RR-TB. The main outcome measure was an unfavorable endpoint (a composite of death, treatment failure, treatment discontinuation, loss to follow-up, and recurrence), and secondary outcome measures included adverse events and serious adverse events. We searched the MEDLINE, EMBASE, Google Scholar, LILACS, and ClinicalTrials.gov databases, from their inception to January 31, 2024, with no limitation as to language or year of publication. The risk of bias was assessed by using the Cochrane risk-of-bias tool, and the quality of evidence was based on the Grading of Recommendations Assessment, Development and Evaluation approach.

**Results:**

A total of 3,668 studies were retrieved; only one (a randomized clinical trial) met the inclusion criteria and was included. In patients with MDR/RR-TB, treatment with the BPaLM regimen, when compared with the standard of care, reduced the risk of an unfavorable outcome (composite, number needed to treat [NNT] = 7); early treatment discontinuation (NNT = 8); adverse events and discontinuation (NNT = 12); and serious adverse events (NNT = 5).

**Conclusions:**

This systematic review of the use of BPaLM in patients with MDR/RR-TB, although it included only one study, showed that BPaLM is more effective than is the standard of care and has a better safety profile. That has major implications for guidelines on the treatment of MDR/RR-TB.

## INTRODUCTION

The tuberculosis epidemic is a major global health problem, and drug-resistant tuberculosis contributes to its mortality worldwide. Globally, an estimated 410,000 people developed multidrug-resistant or rifampin-resistant tuberculosis (MDR/RR-TB) in 2022. However, only approximately two in five were diagnosed and started on treatment.^1^ In addition, there is evidence suggesting that MDR-TB plays an important role in the development of post-tuberculosis lung disease, which is responsible for disability requiring rehabilitation.^2^

With longer treatment regimens, the treatment success rate in patients with MDR-TB is low (approximately 50%). A longer duration of treatment is associated with nonadherence and loss to follow-up. Therefore, the use of shorter treatment regimens that are efficacious and safe could significantly improve treatment success rates in MDR/RR-TB.^3^

The 2022 World Health Organization (WHO) consolidated guidelines from 2022 suggest the use of a 6-month treatment regimen composed of bedaquiline, pretomanid, linezolid (600 mg), and moxifloxacin (BPaLM) rather than the 9-month or longer (typically 18-month) regimens for MDR/RR-TB. In cases of documented resistance to fluoroquinolones, the same regimen but without moxifloxacin (the BPaL regimen) should be used.^4^ Therefore, the objective of this systematic review was to evaluate the available evidence in favor of using the BPaLM regimen for 6 months, compared with other regimens, in patients with MDR/RR-TB.

## METHODS

This systematic review adhered to the guidelines established by the Preferred Reporting Items for Systematic Reviews and Meta-Analyses, and the study protocol conformed to the Grading of Recommendations Assessment, Development, and Evaluation (GRADE) framework.^5^ Because we did not include individual patient data and all data used in the analysis had previously been published, no institutional review board approval was required. The intervention of interest was the use of the BPaLM regimen in patients with pulmonary MDR/RR-TB. The Patients of interest, Intervention to be studied, Comparison of interventions, and Outcome of interest framework was as follows: Patients-adults with pulmonary MDR/RR-TB; Intervention-BPaLM regimen; Comparison-with other regimens; and Outcomes-an unfavorable outcome (a composite of death, treatment failure, treatment discontinuation, loss to follow-up, and recurrence), each of the outcomes in the composite measure, adverse events, and serious adverse events. Treatment failure was defined as need to discontinue or permanently replace at least two treatment drugs with a new regimen, because of adverse events or lack of conversion by the end of the intensive phase; because of bacteriological reversion (new positive culture) in the continuation phase after conversion to negative; or because of evidence of additional acquired resistance to fluoroquinolones or second-line injectable drugs. Loss to follow-up was defined as a patient whose treatment was interrupted for 2 or more consecutive months. We conducted a comprehensive search for randomized clinical trials and observational comparative studies, without imposing restrictions on the date of publication. The inclusion criteria encompassed studies available in full or with summaries and data in Portuguese, Spanish, English, or Italian. The protocol was registered on the international Prospective Register of Systematic Reviews platform (Protocol no. CRD42024527168). One author developed a search strategy that was revised and approved by the team, selected information sources, and systematically searched the following databases: MEDLINE, EMBASE, Google Scholar, LILACS, and ClinicalTrials.gov. The following search string was applied in the MEDLINE and EMBASE databases: (tuberculosis, multidrug-resistant OR drug-resistant tuberculosis OR MDR tuberculosis OR rifampicin-resistant tuberculosis) AND [(diarylquinolines OR bedaquiline) OR (fluoroquinolones OR moxifloxacin) OR (oxazolidinones OR linezolid) OR (nitroimidazoles OR pretomanid)]. For Google Scholar, LILACS and ClinicalTrials.gov, the search strategy was as follows: (tuberculosis) AND (bedaquiline AND moxifloxacin AND linezolid AND pretomanid). 

**Figure 1:**
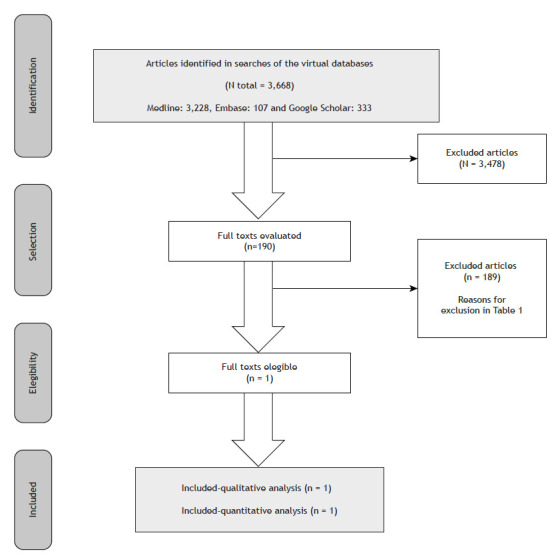
Flow chart of the evidence retrieval and selection process.

Data extraction included information on authorship, publication year, patient characteristics, interventions, absolute numbers for each outcome, and follow-up duration. The extracted values underwent thorough comparison (Figure 1).

The risk of bias assessment utilized the modified Cochrane risk-of-bias tool.^6^ The domains assessed were the randomization process (random sequence generation and allocation concealment), deviations from intended interventions (blinding), missing outcome data, bias in the measurement of the outcome, and selection of the reported results (intention-to-treat analysis, sample size estimation, and early interruption). Risk levels were categorized as low, high, or very high. A meta-analysis was conducted, and the quality of the evidence was assessed by the GRADE approach, the quality of evidence thus being categorized as high, moderate, low, or very low.^7^

The results related to the outcomes are expressed as continuous measures (means and differences of means) or categorical measures (absolute numbers and percentages, risk, or risk differences), with 95% confidence intervals. The analysis was performed with the software Review Manager, version 5.4 (RevMan 5; Cochrane Collaboration, Oxford, UK).^8^

## RESULTS

A total of 3,668 studies were retrieved: 3,228 from MEDLINE; 107 from EMBASE; 333 from Google Scholar; 0 from ClinicalTrials.gov; and 0 from LILACS. After review of the title and abstracts, 3,478 manuscripts were excluded and 190 were selected for full text review. Of those 190 studies, one was included to support this evaluation.^9^. The only study included reported a two-stage, phase 2-3 randomized clinical trial with four intervention arms and one control arm. Therefore, it was not possible to perform a meta-analysis. However, since the study included had two distinct intervention arms with regimens that included BPaL, we used the statistical strategies typically used in meta-analyses to perform an indirect comparison between the results obtained with BPaL, with and without moxifloxacin, and the standard of care. 

The study evaluated involved patients with RR-TB, detected before the start of the intervention. The intervention groups were as follows: arm 1-BPaL, consisting of bedaquiline at a dose of 400 mg daily for 2 weeks followed by 200 mg three times a week for 22 weeks, pretomanid at a dose of 200 mg daily for 24 weeks, and linezolid at a dose of 600 mg daily for 16 weeks followed by 300 mg daily for 8 weeks; and arm 2-BPaLM, with a regimen similar to the BPaL regimen, together with moxifloxacin at a dose of 400 mg daily for 24 weeks. The comparison considered in this analysis was any of the standard WHO-recommended regimens, which could vary according to the country/site, for 44-90 weeks, depending on the regimen used (Table 1).

**Table 1:** Description of the study included in the analysis.

FIRST AUTHOR	YEAR	DESIGN	PATIENTS	INTERVENTION	COMPARISON	OUTCOMES	TIME
Nyang’wa BT	2022	Phase II-III RCT	RR-TB (N = 123)	BPaL: bedaquiline at a dose of 400 mg daily for 2 weeks, followed by 200 mg three times per week for 22 weeks; pretomanid at a dose of 200 mg daily for 24 weeks; and linezolid at a dose of 600 mg daily for 16 weeks, followed by 300 mg daily for 8 weeks	BPaL + clofazimine at a daily dose of 100 mg (or 50 mg if the patient weighed < 30 kg) for 24 weeks or standard of care	Unfavorable outcome (a composite of death, treatment failure, treatment discontinuation, loss to follow-up, recurrence, and adverse events)	72 weeks
			RR-TB (N = 151)	BpaL + moxifloxacin at a dose of 400 mg daily for 24 weeks			

RCT: randomized clinical trial; RR-TB: rifampin-resistant tuberculosis; and BPaL: bedaquiline, pretomanid, and linezolid.

The outcome measures used in our analysis were an unfavorable outcome (composite outcome including death, treatment failure, early treatment discontinuation, loss to follow-up, or recurrence), death, treatment failure, early treatment discontinuation, loss to follow-up, recurrence, and adverse events. A follow-up time of up to 72 weeks was considered (Table 2).

**Table 2:** Outcomes submitted for analysis.

STUDY	UNFAVORABLE OUTCOME		DEATH		EARLY DISCONTINUATION		RECURRENCE		ADVERSE EVENTS (DISCONTINUATION)		SERIOUS ADVERSE EVENTS		LOST TO FOLLOW-UP	
	(n/N)		(n/N)		(n/N)		(n/N)		(n/N)		(n/N)		(n/N)	
	BPaL(M)	SOC	BPaL(M)	SOC	BPaL(M)	SOC	BPaL(M)	SOC	BPaL(M)	SOC	BPaL(M)	SOC	BPaL(M)	SOC
BPaL^9^	24/123	39/152	0/123	2/152	18/123	35/152	3/123	0/152	5/123	17/152	15/123	43/152	3/123	2/152
BPaLM^9^	17/151	39/152	0/151	2/152	15/151	35/152	0/151	0/152	5/151	17/152	14/151	43/152	2/151	2/152

BPaL: bedaquiline, pretomanid, linezolid; BPaLM: BPaL + moxifloxacin; and SOC: standard of care.

### Risk of bias

In the study evaluated,^9^ the risk of bias was very high because of a lack of blinding, losses ≥ 20%, the lack of an intention-to-treat analysis, the absence of sample size calculation, and early interruption (Table 3).

**Table 3:** Risk of bias in the study evaluated.^9^

RANDOMIZATION	BLINDED ALLOCATION	DOUBLE BLIND	BLINDED EVALUATOR	LOSSES	PROGNOSTIC CHARACTERISTICS	APPROPRIATE OUTCOMES	ITT ANALYSIS	SAMPLE ESTIMATION	EARLY INTERRUPTION	ADJUSTMENT FOR CONFOUNDERS
Low	Low	High	High	High	Low	Low	High	High	High	Low

ITT: intention-to-treat.

### Results of the analysis by outcome

**Figure 2:**
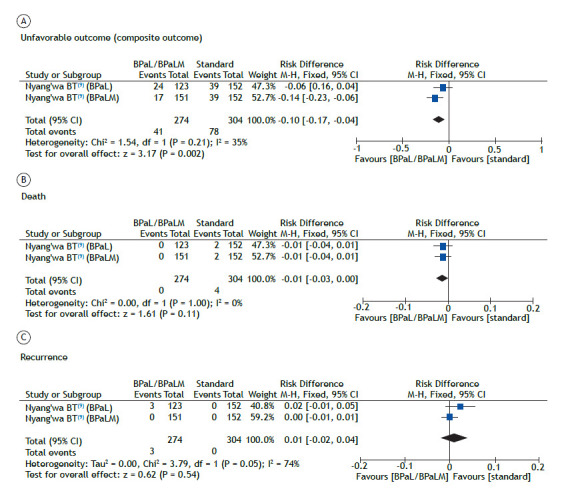
Analysis of the risk of (A) an unfavorable outcome (composite outcome), (B), death, and (C) recurrence, in patients treated with bedaquiline, pretomanid, and linezolid, with or without moxifloxacin (BPaL and BPaLM, respectively).

The risk of an unfavorable outcome was 14% lower (range, 6-23% lower) in the patients treated with the BPaLM regimen than in those receiving the standard of care. The same did not occur in the patients treated with the BPaL regimen (no difference in relation to the standard of care), as illustrated in Figure 2A. The quality of evidence for that risk was categorized as low (Table 4). 

**Table 4:** Quality of evidence regarding the use of bedaquiline, pretomanid, and linezolid, with or without moxifloxacin, in rifampin-resistant tuberculosis.

Certainty assessment				N of patients			Effect			Certainty	Importance	
N of studies	Study design	Risk of bias	Inconsistency	Indirectness	Imprecision	Other considerations	BPaL or BPaLM	Standard of care	Relative (95% CI)	Absolute (95% CI)		
							n/N (%)	n/N (%)				
RISK OF AN UNFAVORABLE OUTCOME												
2	randomized trials	very serious^a^	not serious	not serious	not serious	none	41/274 (15.0)	78/304 (25.7)	RR 0.59 (0.42 to 0.83)	105 fewer per 1,000 (from 149 fewer to 44 fewer)	𑁣𑁣𐊫𐊫 Low	Critical
RISK OF DEATH												
2	randomized trials	very serious^a^	not serious	not serious	not serious	none	0/274 (0.0)	4/304 (1.3)	RR 0.22 (0.03 to 1.89)	10 fewer per 1,000 (from 13 fewer to 12 higher)	𑁣𑁣𐊫𐊫 Low	Critical
RISK OF EARLY DISCONTINUATION												
2	randomized trials	very serious^a^	not serious	not serious	not serious	none	33/274 (12.0)	70/304 (23.0)	RR 0.53 (0.36 to 0.77)	108 fewer per 1,000 (from 147 fewer to 53 fewer)	𑁣𑁣𐊫𐊫 Low	Critical
RISK OF RECURRENCE												
2	randomized trials	very serious^a^	serious^b^	not serious	not serious	none	3/274 (1.1)	0/304 (0.0)	RR 8.64 (0.45 to 165.63)	0fewer per 1,000 (from 0 fewer to 0 fewer)	𑁣𐊫𐊫𐊫 Very low	Major
RISK OF ADVERSE EVENTS (DISCONTINUATION)												
2	randomized trials	very serious^a^	not serious	not serious	not serious	none	10/274 (3.6)	34/304 (11.2)	RR 0.33 (0.17 to 0.65)	75 fewer per 1,000 (from 93 fewer to 39 fewer)	𑁣𑁣𐊫𐊫 Low	Major
RISK OF SERIOUS ADVERSE EVENTS												
2	randomized trials	very serious^a^	not serious	not serious	not serious	none	29/274 (10.6)	86/304 (28.3)	RR 0.38 (0.26 to 0.55)	175 fewer per 1,000 (from 209 fewer to 127 fewer)	𑁣𑁣𐊫𐊫 Low	Major
RISK OF LOSS TO FOLLOW-UP												
2	randomized trials	very serious^a^	not serious	not serious	not serious	none	5/274 (1.8)	4/304 (1.3)	RR 1.41 (0.39 to 5.14)	5 higher per 1,000 (from 8 fewer to 54 higher)	𑁣𑁣𐊫𐊫 Low	Major

a No blinding, no intention-to-treat analysis, losses above 20%, no sample calculation, and interrupted early. ^b^Heterogeneity ≥ 75%

The risk of death in the patients treated with the BPaLM or BPaL regimen did not differ from that calculated for those receiving the standard of care (Figure 2B). The quality of evidence for that risk was categorized as low (Table 4). 

The risk of recurrence in the patients treated with the BPaLM or BPaL regimen did not differ from that calculated for those receiving the standard of care (Figure 2C). The quality of evidence for that risk was categorized as very low (Table 4). 

**Figure 3:**
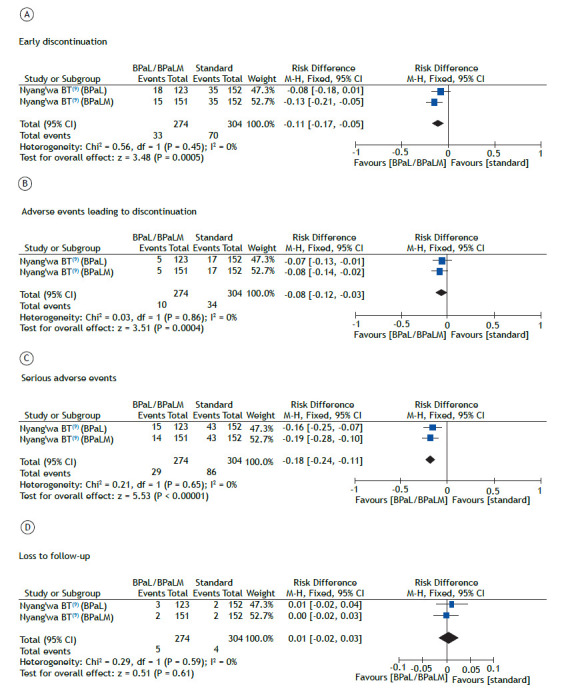
Analysis of the risk of (A) early discontinuation, (B), adverse events leading to discontinuation, (C) serious adverse events, and (D) loss to follow-up, in patients treated with bedaquiline, pretomanid, and linezolid, with or without moxifloxacin (BPaL and BPaLM, respectively).

The risk of early discontinuation of treatment was 13% lower (range, 5-21% lower) in the patients treated with the BPaLM regimen than in those receiving the standard of care. The same did not occur in the group treated with the BPaL regimen (no difference in relation to the standard of care), as shown in Figure 3A. The quality of evidence for that risk was categorized as low (Table 4). 

In comparison with the patients receiving the standard of care, the risk of adverse events leading to treatment discontinuation was 8% lower (range, 2-14% lower) in the patients treated with the BPaLM regimen and 7% lower (range, 1-13% lower) in those treated with the BPaL regimen (Figure 3B). The quality of evidence for that risk was categorized as low (Table 4).

In comparison with the patients receiving the standard of care, the risk of serious adverse events was 19% lower (range, 10-28% lower) in the patients treated with the BPaLM regimen and 16% lower (range, 7-25% lower) in those treated with the BPaL regimen (Figure 3C). The quality of evidence for that risk was categorized as low (Table 4).

The risk of loss to follow-up in the patients treated with the BPaLM or BPaL regimen did not differ from that calculated for those receiving the standard of care (Figure 3D). The quality of evidence for that risk was also categorized as low (Table 4).

### Synthesis of the evidence

In patients with RR-TB:

- Treatment with the shorter BPaLM regimen, when compared with the standard of care, reduces the risk of an unfavorable (composite) outcome (number needed to treat [NNT] = 7); early treatment discontinuation (NNT = 8); adverse events leading to discontinuation (NNT = 12); and serious adverse events (NNT = 5).
- Treatment with the BPaL regimen, when compared with the standard of care, reduces the risk of adverse events leading to discontinuation (NNT = 14); and serious adverse events (NNT: 7).

## DISCUSSION

In this systematic review examining the efficacy and safety of the BPaLM regimen in patients with MDR/RR-TB, we found that treatment with BPaLM reduces the risk of an unfavorable outcome in comparison with the standard of care. In addition, the rates of early treatment discontinuation, adverse events leading to treatment discontinuation, and serious adverse events were lower in the BPaLM group than in the standard-of-care group. Adding moxifloxacin to the BPaL regimen in patients with MDR/RR-TB is recommended because it resulted in a lower risk of an unfavorable outcome and of early treatment discontinuation, as well as to a greater reduction in the risk of adverse events.

In the study evaluated in this systematic review,^9^ 11% of the patients in the BPaLM group and 48% of those in the standard-of-care group evolved to at least one of the outcomes included in the composite primary outcome measure (unfavorable outcome). The per-protocol analysis showed that at least one of those outcomes occurred in 4% of the patients in the BPaLM group and in 12% of those in the standard-care group. Although the fact that only one randomized trial has been published on the subject somewhat limits our certainty around this issue, it was a pragmatic trial, which increases generalizability, its result has already changed practice, and this regimen is now recommended by the WHO.^4^ Reasons for the paucity of randomized clinical trials on the subject include the fact that for many decades no new drugs were approved to treat tuberculosis (bedaquiline was licensed in 2012) and a lack of funding for trials with expensive drug regimens in low- and middle-income countries, where MDR-TB is more common. Further evidence supporting its efficacy include the fact that the results are similar to those of two other studies,^10^ ^,^ ^11^ involving shorter BPaL regimens in patients with extensively drug-resistant tuberculosis, that demonstrated a successful outcome in 84% and 93% of the patients, respectively. Conradie et al.^11^ conducted a randomized trial of treatment for highly drug-resistant tuberculosis with bedaquiline and pretomanid, together with linezolid at two different doses, each with two different durations. All four treatment groups had favorable outcomes in the vast majority of patients (84-93%), the regimen with the best risk-benefit ratio being the one in which linezolid was used at a dose of 600 mg for 26 weeks.

Other shorter regimens have also been shown to be associated with successful outcomes in most patients. The STREAM trial^12^ compared a short regimen (of 9-11 months) including moxifloxacin at a high-dose with a long, WHO-recommended regimen (of 20 months), for the treatment of patients with RR-TB. In the short regimen group, 78.8% of the patients had a favorable outcome, demonstrating that it was noninferior to the long regimen. In a retrospective cohort analysis on patients with RR-TB treated with a standardized all-oral short regimen (including bedaquiline and linezolid as the core drugs), treatment success was achieved in 75.2% of the patients.^13^ The final analysis of the trial included in our review corroborates, with improved precision, the noninferiority of the BPaLM regimen when compared with the standard of care.^14^ Recently, the regimen of bedaquiline, pretomanid, moxifloxacin, and pyrazinamide showed promising results.^15^

Our study has some limitations. First, only one study met the inclusion criteria and was included for analysis. Therefore, a meta-analysis could not be performed. However, we evaluated each intervention arm that included BPaL, in comparison with the standard of care. More studies are needed in order to confirm these findings. Shorter regimens using newer antituberculosis drugs are relatively new, and there are several combinations of drugs that can be used. Therefore, this meta-analysis may need to be updated as more clinical trials of this treatment strategy are published. Second, the randomized trial evaluated was interrupted early for efficacy after recruitment of 75% of the planned sample, which could have resulted in an overestimation of the treatment effect.^16^ Finally, the standard-of-care regimens varied across studies and could be updated as the WHO makes new treatment recommendations, although all regimens were in line with current WHO recommendations. Despite these limitations, the population included in the study was diverse, including HIV-coinfected patients and patients with fluoroquinolone-resistant tuberculosis, covering a broad spectrum of cases of RR-TB. 

In conclusion, this systematic review of the use of BPaLM in patients with RR-TB found that treatment with this regimen is more effective and has a better safety profile in comparison with the standard of care. This finding has major implications for the development of new treatment guidelines that can contribute to better outcomes in tuberculosis treatment worldwide. 
